# A Pain in the Hip: Sexually Transmitted Septic Arthritis Diagnosed With Bedside Ultrasound in the Emergency Department

**DOI:** 10.7759/cureus.98653

**Published:** 2025-12-07

**Authors:** Ian D Storch, Justin Nippert

**Affiliations:** 1 Emergency Medicine, University of Florida College of Medicine – Jacksonville, Jacksonville, USA

**Keywords:** disseminated gonococcal arthritis, disseminated gonococcal infection, hip effusion, septic arthritis, septic arthritis of hip

## Abstract

Disseminated gonococcal infection (DGI) is a recognized but uncommon complication of a sexually transmitted infection (STI). Some physicians and advanced practice providers may have a misunderstanding of the onset of DGI and think of it as a later manifestation of a gonococcal infection similar to syphilis, another common sexually transmitted infection. This is a case report of a patient tested for STIs 16 days prior to his presentation with negative results. However, he presented to the emergency department again with right hip pain one day after being seen for a urinary tract infection. Despite the short amount of time between negative test results and repeat emergency department visits, keeping a broad differential diagnosis allowed for the correct diagnosis to be made. This case report provides a better understanding of the onset of gonococcal septic arthritis, discusses the proper workup, differences in treatment compared to some other causes of septic arthritis, and the likely prognosis of gonococcal septic arthritis.

## Introduction

Septic arthritis of the hip, or any joint, is a medical emergency requiring prompt recognition and treatment. It can lead to rapid joint destruction and significant morbidity if not diagnosed and treated in a timely manner. While many bacteria can cause septic arthritis, *Neisseria gonorrhoeae* is a common bacterial cause of septic arthritis in sexually active patients. This case report discusses a patient with gonococcal septic arthritis of the hip, highlighting the clinical presentation, diagnostic challenges, and management strategies.

There are at least 600,000 cases of gonorrhea each year in the United States [1]. Depending on how robust the local healthcare infrastructure is and the access to healthcare in a region, the training level of the medical care provider who is initially evaluating these patients can vary widely. These patients can present to public health department clinics, primary care clinics, OB/GYN clinics, and emergency departments, among other health care facilities. The sheer number of cases of gonorrhea each year, and the clinics in which they present, underscores the importance of maintaining clinical vigilance for complications.

*Neisseria gonorrhoeae* is a gram-negative *diplococcus* primarily known for causing localized genitourinary infections. Normally, patients will present with symptoms within 2-5 days at the primary mucosal site of infection [2]. However, in a subset of cases, it can disseminate hematogenously, leading to disseminated gonococcal infection (DGI). Dissemination is rare, estimated to be < 1% of cases of gonorrhea [3]. However, some older literature suggested a 1-3% of cases of gonorrhea would present with dissemination [2]. It is unclear if this decrease is due to more accurate and timely testing for the disease, more public awareness, or easier access to treatment. When it does spread, it can happen as quickly as 2-3 weeks after infection at the primary site [3]. Disseminated gonococcal infections most commonly present with a triad of tenosynovitis, polyarthralgia, and dermatitis, or as septic arthritis. This case report describes the timeline, workup, and treatment of a patient diagnosed with septic arthritis from a disseminated gonococcal infection.

## Case presentation

A 49-year-old male presented to the ED for right hip and knee pain for one day. He had a past medical history of hypertension, epilepsy, alcohol abuse, and chronic pancreatitis, and he regularly smoked tobacco cigarettes. According to the chart review, the patient was seen 16 days ago for urinary symptoms and had a urine analysis that did not show signs of blood or infection and negative STI testing. However, the patient was also seen one day prior to our presentation, and this time was diagnosed with a urinary tract infection, thought to be causing pyelonephritis since he was having right flank pain. He was discharged on a 10-day course of cephalexin. STI testing from one day prior had not resulted yet.

The patient returned the next day due to new right hip and knee pain. The hip and knee pain were present when he woke up, and it rapidly progressed to the point that he could no longer ambulate on the right leg over the course of a couple of hours. He stated he could not afford the medication from the previous visit and therefore never picked it up.

He denied any acute or prior trauma to the leg and denied any prior surgeries to his lower extremities. He denied having a rash, tenderness to touch, numbness, weakness, fever, chills, or swelling. He was able to walk normally the previous day, but starting this morning, he has had significant pain with range of motion. He preferred a slightly flexed position while lying down on his back. Denied IV drug use and also denied sexual intercourse in the past year. He denied oral, vaginal, or anal penetration over that time span. Last alcohol use was half a can of beer that morning, and they denied a history of falling while intoxicated.

His vital signs revealed a temperature of 36.7°C, a pulse of 93, a blood pressure of 163/103, a respiratory rate of 16, and a SpO2 of 98%, and he weighed 72 kg.

From the doorway, our initial physical exam did not reveal a patient in acute distress. He had no visible rash, scleral icterus, or jaundice. On further examination, he had a normal cardiovascular and pulmonary exam with no murmurs or abnormal breath sounds. He had a benign abdominal exam but did have some right costovertebral angle tenderness. His genital exam did not reveal any rash, ulceration, or warts. He did have right inguinal lymphadenopathy. As for his extremity exam, the passive range of motion of the right ankle and knee was intact. However, he had significant pain with both active and passive internal and external rotation of the right hip.

His workup included repeat STI testing, blood work, a bedside ultrasound of the hip with a plan for CT or MRI, and starting IV antibiotics. His urine sample from the day before had been sent for a urine culture. Although he did not have a fever, he was diagnosed with pyelonephritis on the day prior. However, the patient had failed to pick up antibiotics after his discharge home from that visit. The inguinal lymphadenopathy exam could have been a localized reaction to the patient's urinary tract infection and pyelonephritis, but with the hip pain and limited range of motion, septic arthritis was suspected. Initially, disseminated gonorrhea would seem unlikely since he recently had negative STI testing. However, septic arthritis from an STI or other causative bacterial infections was not excluded and remained likely given the overall presentation.

The patient's previous urine analysis and STI testing results were compared, revealing that the previous negative value was now positive (Tables 1, 2). The STI testing during this encounter was actually performed directly from the aspirated effusion and not a urine or mucosal test. The patient's blood work was notable for mild elevations in white blood cells (WBC), C-reactive protein (CRP), and erythrocyte sedimentation rate (ESR) (Tables 3, 4).

**Table 1:** Urine Analysis from the Day Before Our Encounter WBC: White Blood Cells

	Result
Color	Yellow
Clarity	Hazy
pH	6
Specific Gravity	1.009
Bilirubin	Negative
Blood	Small
Glucose	Negative
Nitrite	Negative
Leukocyte Esterase	Large
White Blood Cells	143
Ketones	Negative
Red Blood Cells	6
Squam Epithelial	1
Bacteria	Rare
Hyaline Casts	3
Mucus	Rare
Urobilinogen	Normal
WBC Clumps	1+
Protein	Negative

**Table 2:** STI Test Results Spanning 16 Days Initial results are from a urine sample 16 days prior to our encounter: all results are negative. The second set of results is from a urine sample one day prior to our encounter: all results are negative. The third set of results are from the hip aspirate on the day of our encounter: positive for *Neisseria gonorrhoeae*. STI: Sexually Transmitted Infection

Organism	Result
Neisseria Gonorrhoeae	Negative
Chlamydia Trachomatis	Negative
Mycoplasma Pneumoniae	Negative
Neisseria Gonorrhoeae	Positive
Chlamydia Trachomatis	Negative
Mycoplasma Pneumoniae	Negative
Neisseria Gonorrhoeae	Positive
Chlamydia Trachomatis	Negative
Mycoplasma Pneumoniae	Negative

**Table 3:** CBC w/ Differential CBC: Complete Blood Count, MCV: Mean Corpuscular Volume, MCH: Mean Corpuscular Hemoglobin, MCHC: Mean Corpuscular Hemoglobin Concentration, RDW: Red Cell Distribution Width

	Result	Reference Range
White Blood Cells	13.33	4.5-11 thousand/cubic mm
Red Blood Cells	3.21	4.2-5.4 x 10e6/µL
Hemoglobin	11.1	12.0-16.0 j/dL
Hematocrit	31.7	37-47 %
MCV	98.8	82-101 fL
MCH	34.6	27-34 pg
MCHC	35	31-36 g/dL
RDW	13.2	12.0-16.1 %
Platelet Count	117	140-440 x 10e3/µL
Neutrophils %	70.6	34-73 %
Immature Granulocytes	0.7	%
Lymphocytes %	15.4	25-45 %
Monocytes %	12.8	%
Eosinophils %	0.1	%
Nucleated Red Blood Cells %	0	0-1 %
Basophils %	0.4	Ref Range Not Established %
Neutrophils Absolute	9.43	1.40-7.50 x 10e3/µL
Absolute Immature Granulocytes	0.09	0-0 x 10e3/µL
Lymphocytes Absolute	2.05	0.9-3 x 10e3/µL
Monocytes Absolute	1.7	0.2-0.9 x 10e3/µL
Eosinophils Absolute	0.01	0.0-0.5 x 10e3/µL
Basophils Absolute	0.05	0.0-0.1 x 10e3/µL
Absolute Nucleated RBC Count	0	10*9/L
Burr Cells	Occasional	none
Ovalocytes	Occasional	none
Poikilocytes	Slight	none
Target Cells	Occasional	none

**Table 4:** Inflammatory Markers

	Result	Reference Range
Erythrocyte Sedimentation Rate	62	0-15 mm/hr
C-Reactive Protein	17.8	0.1-2.8 mg/L

**Figure 1:**
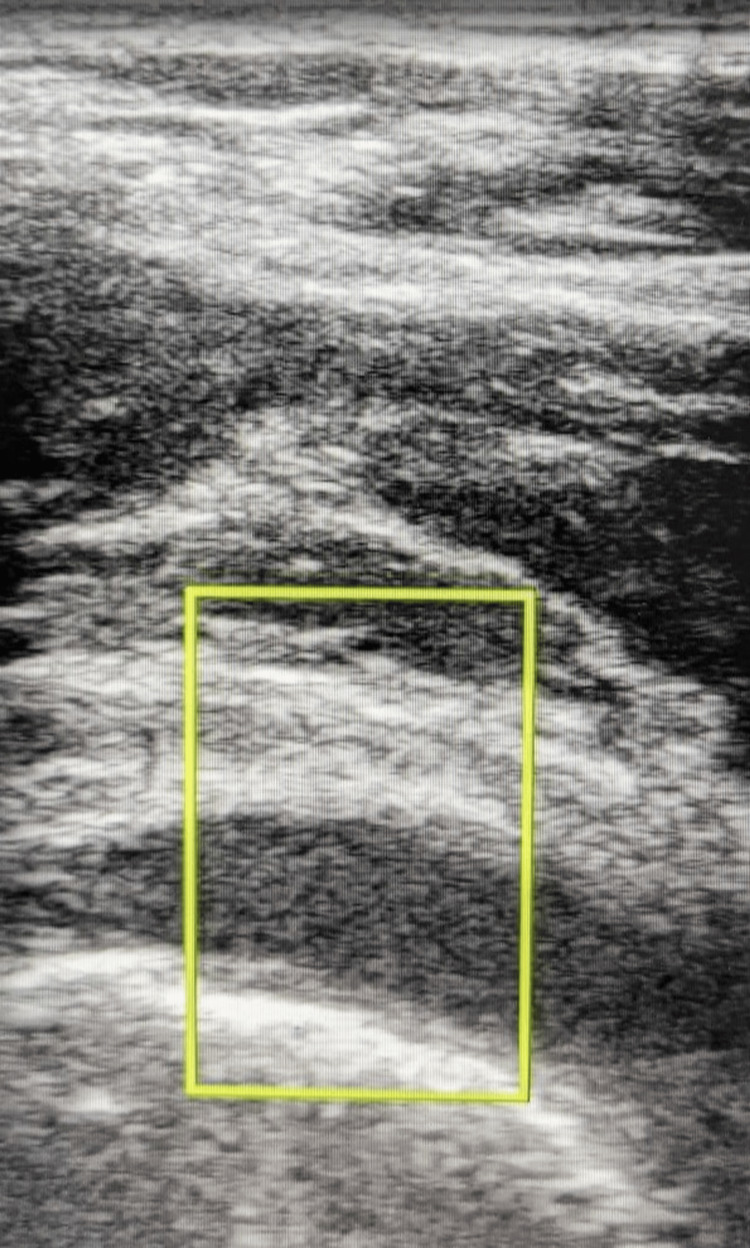
Hip Effusion Seen on Ultrasound The effusion is the hypoechoic (darker grey and black area) seen just above the hyperechoic (bright white) curvature of the femoral head toward the bottom third of the image. Color Doppler, which would be found inside the yellow box, was used over the effusion area. Since there was no color flow seen, it was less likely to be a hypoechoic area due to vascular etiology. There was also no evidence of color flow immediately surrounding the hypoechoic area that might suggest a rim-enhancing abscess.

**Figure 2:**
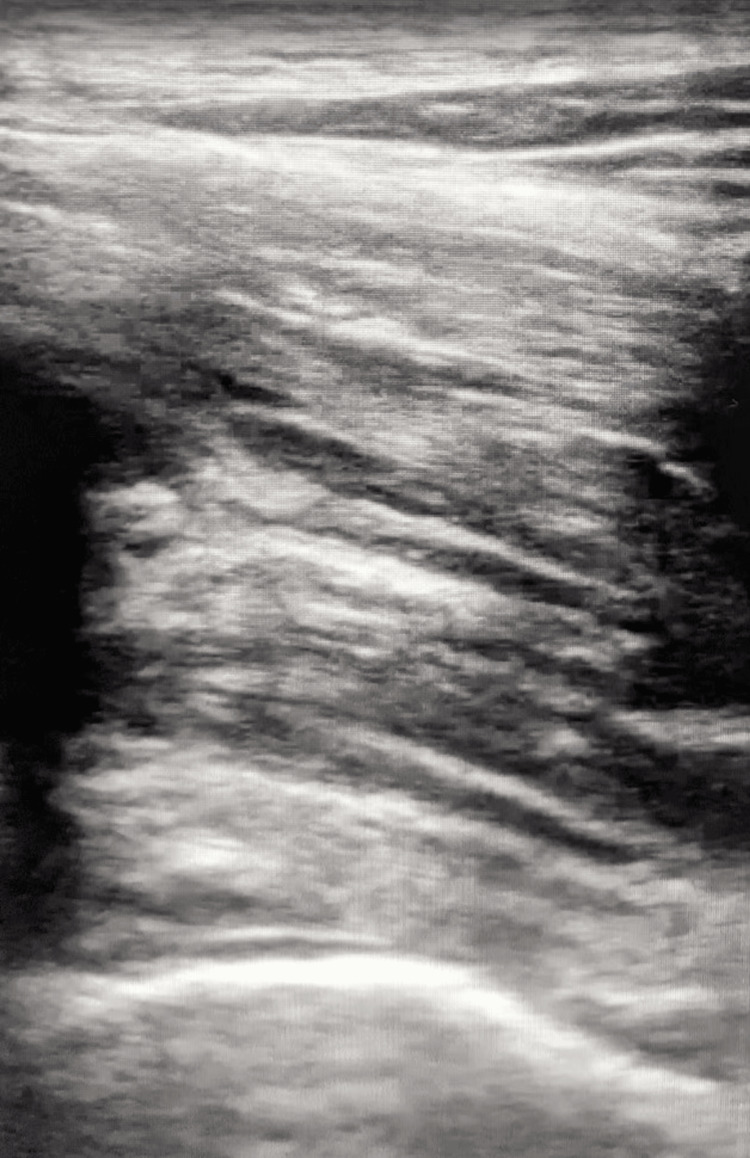
Hip Effusion After Arthrocentesis The previously seen hypoechoic area above the hyperechoic curvature of the femoral head over the bottom third of the screen is no longer seen after performing the arthrocentesis.

**Figure 3:**
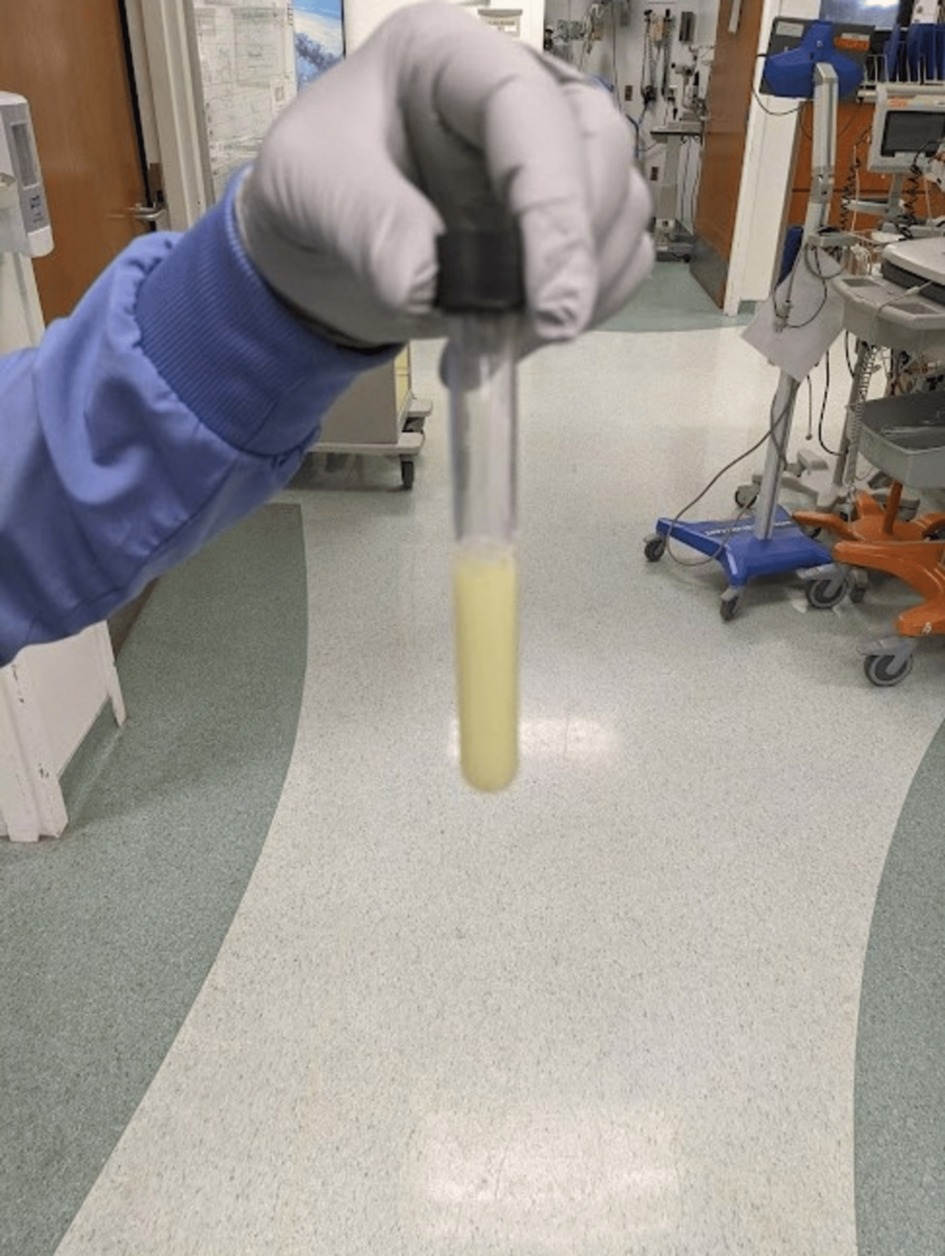
Purulent Synovial Fluid Cloudy, purulent synovial fluid aspirated from the right hip joint.

Prior to the patient getting a CT or MRI, a bedside ultrasound was performed by the treating emergency medicine physician to evaluate for a joint effusion. An arthrocentesis would be diagnostic if an effusion were present and an aspirate sample could be obtained (Figure 1). The ultrasound images revealed a joint effusion, which would not have been expected in the right hip of this patient with no acute injuries to the hip or any history of arthritis. The effusion was drained by the emergency medicine physician using ultrasound guidance until most of the effusion was aspirated (Figure 2). Draining the fluid helped make a diagnosis and was also therapeutic, as the patient's degree of pain and range of motion improved after the procedure. The aspirated material was thick and cloudy, unlike normal synovial fluid (Figure 3).

**Figure 4:**
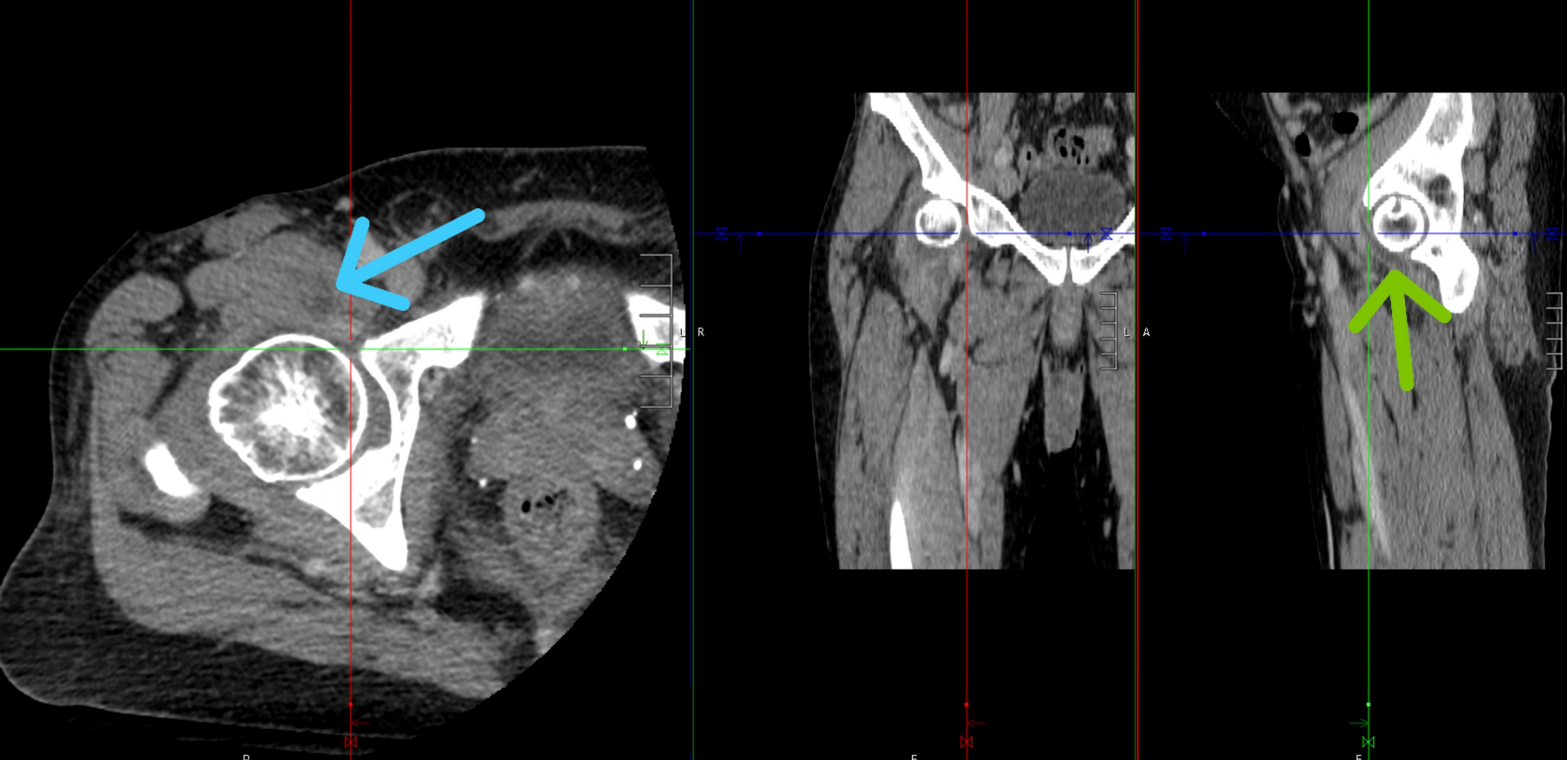
CT of Right Hip The blue arrow denotes a region of fluid in the musculature from the arthrocentesis anesthetic. The green arrow and the red, green, and blue horizontal and vertical triangulation lines pinpoint the small effusion around the femoral head. CT: Computed Tomography

The visual appearance of the aspirate was suggestive of a bacterial septic arthritis, so IV antibiotics were continued, and the synovial fluid was sent for cell count, Gram stain, crystal analysis, and culture (Table 5). A CT abdomen/pelvis and CT of the right hip were performed to further exclude any abscess formation, other areas of dissemination, or complications of pyelonephritis or a presumed STI. The CT revealed a small remaining right hip effusion, without osseous erosions, but no other obvious complications (Figure 4).

**Table 5:** Right Hip Effusion Arthrocentesis

Synovial Fluid day of encounter:	
Appearance	Hazy
Color	Yellow
Red Blood Cell Count	3,000
White Blood Cell Count	61,680
Crystals	None Seen

## Discussion

The clinical presentation of gonococcal septic arthritis is often subtle, which can delay diagnosis. Patients typically present with monoarticular or oligoarticular arthritis accompanied by fever, chills, and malaise. A distinguishing feature from other forms of septic arthritis is the frequent presence of dermatitis, tenosynovitis, and migratory arthralgias prior to joint involvement [2]. Classic symptoms of urethritis may not be present. Careful history taking, particularly including sexual history, is essential. This case had some common themes in that the patient's diagnosis was initially pyelonephritis based on a urine sample that appeared to have an infection, and the patient was having right-sided abdominal or flank pain. The urine infection was likely caused by his STI, and the right-sided pain may have been migratory arthralgias prior to localizing to the right hip. The sexual history was likely unreliable given the final outcome of this case.

Gonococcal septic arthritis represents a manifestation of DGI, which occurs in a minority of patients with untreated mucosal N. gonorrhoeae infection. The pathogenesis involves hematogenous spread from a primary mucosal site (genitourinary, pharyngeal, or rectal) to the synovium. Bacteremia permits gonococcal seeding of synovial tissue, triggering an inflammatory cascade characterized by synovial hyperplasia, neutrophil infiltration, and purulent effusion. The hip is a particularly destructive site due to its deep location and limited collateral circulation, which impairs both immune response and antibiotic penetration. Delayed diagnosis can lead to worse outcomes, including chronic arthritis, decreased mobility, and decreased quality of life that can follow.

Septic arthritis of the hip due to gonorrhea often presents acutely with severe hip pain, restricted range of motion, fever, and an antalgic gait or inability to bear weight [3]. Patients with DGI may have minimal or absent genitourinary symptoms, complicating diagnosis. While the knee and wrist are more frequently affected, hip involvement can result in rapid joint destruction if untreated.

Joint aspiration remains the gold standard of diagnosis. Synovial fluid typically demonstrates a purulent appearance with WBC counts >50,000 cells/µL and neutrophil predominance, though lower counts may occur early in infection. Gram stains are often negative, and cultures may fail due to the organism’s fastidious nature, making nucleic acid amplification testing (NAAT) the preferred diagnostic modality. Cultures from potential mucosal reservoirs should also be obtained. Blood cultures, while often negative, should be performed. Imaging may reveal an effusion or early joint destruction. MRI can detect subtle inflammatory changes. Ultrasound may reveal a joint effusion and may aid in arthrocentesis. 

Timely intervention is critical to prevent irreversible damage. Treatment consists of antibiotic therapy combined with joint drainage. Empiric antibiotic coverage should take into consideration both *N. gonorrhoeae* and *Staphylococcus aureus* since *Staphylococcus aureus* is a common cause of septic arthritis. Ceftriaxone remains the drug of choice, typically given intravenously for 10-14 days, with azithromycin added to address potential *C. trachomatis* co-infection and mitigate resistance. Therapy may transition to oral agents based on clinical response and culture results [4,5]. The joint effusion was aspirated a second time during hospitalization. After repeat imaging did not show further accumulation, and the patient's clinical status showed improvement in range of motion, he was able to be transitioned to an oral antibiotic treatment.

Drainage of purulent material is an important step in managing septic arthritis. Unlike some other causes of septic arthritis, washout in the operating room or a surgical approach is not as common [6]. Serial needle aspiration is often sufficient for gonococcal cases, given the typically lower viscosity of the effusions. Arthroscopic or open surgical drainage is indicated when aspiration is inadequate, when loculations are present, or when there is concern for cartilage destruction.

With prompt recognition and therapy, the prognosis of gonococcal septic arthritis is generally favorable, especially when compared to non-gonococcal causes of septic arthritis. However, hip involvement increases the risk of long-term complications such as osteonecrosis, chronic osteomyelitis, or permanent dysfunction if diagnosis and treatment are delayed due to the unique blood supply to the joint and the depth of the joint, sometimes limiting complete aspiration of all infectious material.

## Conclusions

Our case report highlights the timing of disseminated gonococcal septic arthritis, the utility of bedside ultrasound used by an emergency physician to aid in diagnosis by obtaining a synovial sample, and the treatment of gonococcal septic arthritis. Septic arthritis of the hip is rare, but gonococcal septic arthritis of any joint can potentially be devastating if left untreated. A high index of suspicion is warranted for all sexually active patients, regardless of the sexual history they self-report. Synovial fluid analysis, STI testing, and the initiation of IV antibiotic therapy with ceftriaxone are the most important early steps of management. The patient may go on to have repeat arthrocentesis procedures while admitted to the hospital as part of their treatment. Surgery is not always indicated, as with other causes of septic arthritis. Gonorrhea continues to have a high prevalence in the United States. The impact of emerging antibiotic resistance on the incidence of DGI remains uncertain. Until then, it is essential that medical providers at diverse clinical settings understand these aspects of diagnosis and treatment in patients with suspected STIs or monoarticular joint pain.
